# The mean platelet volume and atherosclerotic cardiovascular-risk factors in adults with obesity: a systematic review and meta-analysis of observational studies

**DOI:** 10.1186/s40795-022-00541-8

**Published:** 2022-05-16

**Authors:** Bongani Brian Nkambule, Vuyolwethu Mxinwa, Tawanda Maurice Nyambuya, Phiwayinkosi Vusi Dludla

**Affiliations:** 1 School of Laboratory Medicine and Medical Sciences (SLMMS), College of Health Sciences, University of KwaZulu-Natal, Private Bag X54001, Durban, 4000 South Africa; 2 Department of Health Sciences, Faculty of Health and Applied Sciences, Namibia University of Science and Technology, Windhoek, Namibia; 3 Biomedical Research and Innovation Platform (BRIP), Medical Research Council (MRC), Tygerberg, Cape Town, South Africa

## Abstract

**Background:**

Obesity is a major risk factor for atherosclerotic cardiovascular disease (ASCVD) and is associated with altered platelet function. The mean platelet volume (MPV) is a rapid measure of platelet activation and a prognostic marker in patients with cardiovascular disease. However, no meta-analysis on the association between MPV and obesity has been conducted, and the value of monitoring the MPV in patients with obesity remains unclear.

**Objective:**

To provide cumulative evidence on whether the mean platelet volume (MPV) is increased in individuals with obesity and to describe associations between the ASCVD-risk factors and the MPV in individuals with obesity.

**Methods:**

This meta-analysis was prepared following the Meta-analysis Of Observational Studies (MOOSE) guidelines. We searched the PubMed and Embase database from inception until the 31st of March 2021. Studies were included when they reported the mean platelet volume in individuals with obesity and provided a suitable non-obese comparator group. The risk of bias was independently assessed by two reviewers using the Newcastle–Ottawa scale. The primary outcome of the meta-analysis was the MPV, while we considered the atherosclerotic risk profiles as a secondary outcome.

**Results:**

We identified 178 citations through the PUBMED and 255 citations through EMBASE database search. In all, 13 studies met the inclusion criteria. Firstly, we report an increased mean platelet volume in individuals with obesity compared to non-obese individuals (MD 0.79; [95%CI: 0.42 to 1.16], I2 = 93.4%). Moreover, the reported increase in the MPV was inversely associated with the body mass index (Coefficient: -0.57, standard error (SE): 0.18, *p* < 0.001) and directly related to changes in triglyceride levels (Coefficient: 4.99, standard error (SE): 1.14, *p* < 0.001).

**Conclusion:**

This meta-analysis and meta-regression showed an increased MPV in nondiabetic individuals living with obesity. Moreover, the MPV was associated with hypertriglyceridemia, an independent predictor of atherosclerotic cardiovascular disease. Overall, the findings suggest that MPV may be a valuable rapid marker for the monitoring and risk-stratification of individuals with obesity who may be at risk of developing cardiovascular disease.

**Supplementary Information:**

The online version contains supplementary material available at 10.1186/s40795-022-00541-8.

## Introduction

Obesity affects more than a third of the population living in low-to-middle- and high-income countries. It has also emerged as a significant healthcare challenge affecting children and adults [1]. On a global scale, over 650 million adults are obese, with sex and ethnic disparities in the prevalence of obesity [2]. The prevalence of obesity ranges from 3.7% to 7.0% in Asia [1] and as high as 39.2% in the United States of America [3]. Obesity is one of the most prevalent comorbid diseases and an independent risk factor of significant non-communicable diseases like type 2 diabetes, cardiovascular disease, and malignancies. Notably, the prevalence of obesity is commonly estimated using the body mass index (BMI), and several BMI cut-off points exist for children(5–10 years), adolescents(11 to 18 years) and adults (> 18 years) [4]. The WHO BMI cut-off points are known to underestimate the risk of obesity in Asian populations [5]. There are currently three major classification systems used to assess overweight and obesity, and these include the International Obesity Task Force (IOTF)[6], the United States Centers for Disease Control and Prevention [7] and the World Health Organization (WHO) criteria [8]. 

A previous meta-analysis of Mendelian randomisation studies provided evidence of obesity as an independent predictor of coronary artery disease and showed no association with the risk of strokes [9]. A hypercoagulable state in patients with obesity is primarily due to platelet dysfunction, increased thrombin formation, and impaired fibrinolysis [10]. Activated platelets play a pivotal role in inflammation and the development of atherosclerotic cardiovascular disease (ASCVD). The statistical models used to estimate the 10-year ASCVD-risk of individuals between the ages of 40 to 79 years are based on risk factors which include age, total cholesterol, high-density lipoprotein cholesterol, treated or untreated systolic blood pressure levels, diabetes mellitus status, and their smoking status [11]. The basal platelet activation and reactivity levels predict patient outcomes following antiplatelet therapy [12]. Since the therapeutic adjustment of antiplatelet drug dosing is dependent on weight and platelet responses, in a clinical setting, point of care testing is crucial for optimal patient management [12, 13].

A clinical dilemma arises in patients with obesity, as obesity is associated with impaired platelet responses. The mean platelet volume (MPV) is a convenient measure of platelet activation that is routinely available and can be utilised in an inpatient and outpatient setting [14]. In a previous meta-analysis, *Chu *et al., showed the potential value of the MPV as a prognostic marker in patients with CVD [14]. The MPV provides rapid platelet activation and thrombotic risk measurement, as the MPV correlates with platelet size, activation, and increased aggregation [15]. Notably, activated and younger pro-thrombotic platelets are associated with a higher MPV [16]. Although the clinical relevance and interpretation of the MPV in individuals with obesity remains uncertain, controversial findings of increased [15, 17–23] and comparable [24] MPV in obese compared to lean individuals, have been reported in observational studies.

No meta-analysis on the MPV in individuals with obesity has been conducted to date. The value of the MPV as a determinant of thrombotic risk in obese patients may offer a practical and cost-effective surrogate of platelet activation in obese patients who may be at risk of developing ASCVD. This meta-regression and meta-analysis were conducted to estimate the MPV in individuals with obesity by pooling data reported in studies evaluating the MPV in obese populations. The emphasis of the current study is to provide cumulative evidence on whether the MPV is increased in individuals with obesity and to describe associations between the ASCVD-risk factors and the MPV in individuals with obesity.

## Methods

The study protocol was not registered, and therefore, no registration number has been allocated. This systematic review and meta-analysis was reported in accordance with the Meta-analysis Of Observational Studies in Epidemiology (MOOSE) guidelines [25]. All data searches and collection took place between February 2020 and April 2021. There was no patient-level data collected, and the protocol required no informed consent or institutional approval as only publicly available data was accessed. The search strategy was designed to retrieve published studies that aim to address the following research questions;

1. Is the mean platelet volume increased in individuals with obesity?
2. Are there any associations between the atherosclerotic cardiovascular-risk factors and the MPV?

### Information sources and search strategy

We conducted a systematic search using medical subject headings (MesH) on MEDLINE and EMBASE search headings (Emtree) on the EMBASE database, from inception until the 31^st^ of March 2021. The search was independently conducted by two reviewers (BBN and VM) using the PUBMED and OVID interface. The reviewers used the following MeSH terms and text words to retrieve the relevant studies; “Obesity” AND “Blood Platelets” OR “Thrombocytes” OR “Platelet count” OR “Mean Platelet Volume” OR “Plateletcrit” OR “Platelet Distribution Width”. The search strategy was adapted for the ISI Web of Science, the international prospective register of systematic reviews (PROSPERO), Cochrane Collaboration, and the Joanna Briggs Institute (JBI) protocol registry. We restricted the search to human clinical studies with no language restrictions applied. Additional scanning of the bibliographies of the retrieved studies was used to augment the retrieval strategy and ascertain that no relevant citations were inadvertently filtered out through the database search. A detailed description of the search strategy used on the MEDLINE database using the PUBMED search engine is provided on supplementary file 1 (Table 1S), and the Embase data search using the OVID interface is presented in supplementary file 1 (Table 2S).

### Eligibility criteria

The studies were included based on the fulfilment of the following eligibility criteria:

#### Population

1. For adult participants, we included nondiabetic individuals living with Class I obesity (BMI 30.0-34.9 kg/m2 ) and class II obesity (BMI 35 35.0-39.9 kg/m2). Whereas for Asian populations, adults with a BMI ≥ 25 kg/m2 were considered as living with obesity.
2. For children and adolescents, we included nondiabetic individuals living with Class 1 obesity (BMI ≥ 95th percentile to <120% of 95th percentile for age and sex) and class II obesity (BMI ≥ 120% to < 140% of 95th percentile or BMI ≥ 35 kg/m2).

#### Interventions and comparators

There were no specific interventions considered for this study. While the comparator included non-obese individuals (BMI < 25.5).

### Outcomes

The primary outcome included the difference in mean platelet volume. The secondary outcome included atherosclerotic cardiovascular disease (ASCVD) risk markers, such as BMI; systolic blood pressure; diastolic blood pressure; high-density lipoprotein and low-density lipoprotein cholesterol.

### Study design and selection

In this systematic review and meta-analysis, we included observational studies. We included studies that reported the mean platelet volume as one of the study outcomes. Studies that were case studies, reviews, letters to the editor, animal models or cell lines were excluded.

### Data collection process and data items

Two reviewers (BBN and VM) independently extracted detailed study-level participant data using the adapted Cochrane Consumers and Communication Review Group data extraction sheet for included studies template [26]. The data items included the names of the authors; year of publication; sample size; participant age, gender, and BMI; primary outcome measures (MPV) and secondary outcome measures (Systolic blood pressure, diastolic blood pressure, glucose, low-density lipoprotein-c, high-density-lipoprotein-c, triglycerides). A third reviewer (PVD) was consulted for arbitration in instances of discrepancies in the extracted data items.

### Assessment of study quality and confidence in the cumulative evidence

The Newcastle–Ottawa tool was used to assess the quality of the included studies [27]. The tool assesses the risk of bias within the following domains; selection of study groups, comparability, and the outcome. Two authors (VM and KM) independently graded the included studies, which were classified as low-quality (score between 0–4), Moderate-quality (score of 5–6), and high-quality (score > 7). Two reviewers (TNM and VM) used the Grading of Recommendations Assessment, development, and Evaluation (GRADE) approach to evaluate the quality of the cumulative evidence [28]. The GRADE tool consists of three domains: consistency, directness, precision, and publication bias. Based on these domains, the certainty of the evidence for each outcome was rated as low, moderate, and high.

### Statistical analysis

We computed and estimated the standardised mean difference (SMD) and 95% confidence interval (CI) between obese and non-obese individuals for each included study. The reported probability values (p-values) were also pooled using Edington’s additive method [29]. We then estimated the effect size using the random-effects model when the levels of statistical heterogeneity were substantial (I^2^ > 50%), and we used a fixed-effects model when the levels of statistical heterogeneity were low (I^2^ < 40%) [30]. The between-study variance was assessed using the T^2^ and I^2^ statistics. Since platelet indices may be affected by geographical variations, a post hoc subgroup analysis was performed based on the reported study location. Moreover, publication bias was assessed using visual inspection of funnel plots and the Egger’s test. All statistical analyses were performed using SATA 16.0 (StataCorp LP, College Station, TX, USA). The risk of bias plots were prepared using the robvis tool [31].

### Meta-regression analysis

We performed a random-effects-weighted meta-regression analysis to assess the association between BMI and mean platelet volume. Due to known changes in MPV levels due to age and BMI, a meta-regression was used performed to explore the potential interaction between age and the MPV. The residual maximum likelihood method was used to calculate the between-study component of variance.

## Results

### Characteristics of included studies

**Fig. 1:**
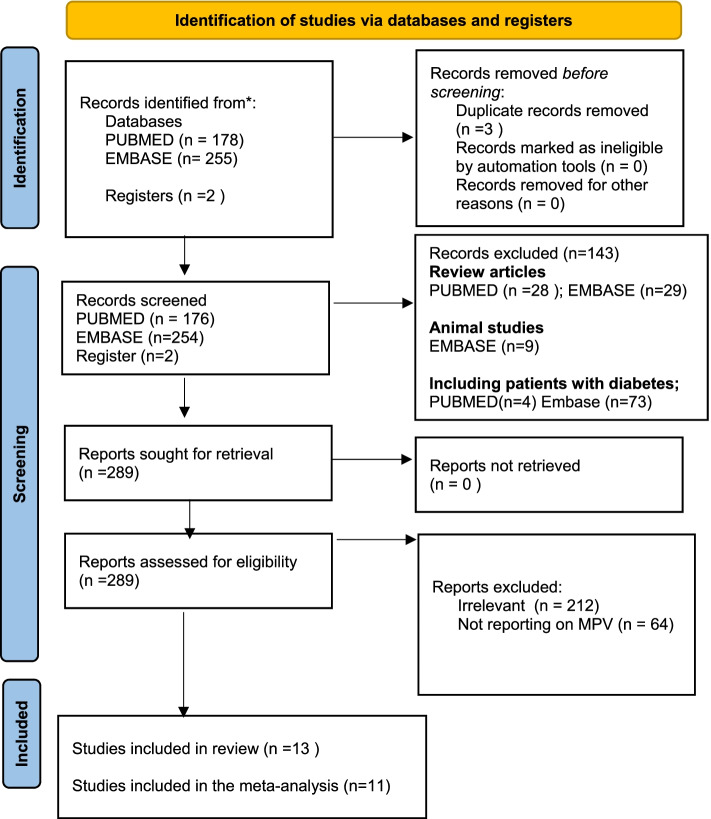
PRISMA flow diagram illustrating the study selection process

We identified 178 citations through the PUBMED, 255 through EMBASE database search and two citations through searching registries. We screened all retrieved citations, and after excluding duplicates, a total of 430 studies were eligible for full-text screening. In all, 13 studies were included in the qualitative synthesis, whereas eleven studies were included in the meta-analysis (Fig. 1). Notably, the included studies were all cross-sectional studies comprised of studies from 3 different countries, which included; Turkey (*n* = 9)(16,17,18,19,20,22,23,24), Poland (*n* = 1) [21], Malaysia (*n* = 1) [32], China (*n* = 1) [33], and Brazil (*n* = 1) [23] (Table 1). The meta-analysis comprised of 814 patients with obesity and 598 non-obese individuals. Only six studies(17,18,19,20,21,22); reported on the mean age and 6 studies [18–23] reported on the mean BMI of obese participants (Table 2). The mean age of the included obese participants was 44.03 ± 18.01, while the mean age of the control participants with normal body weights was 41.67 ± 17.93. Overall, the individuals with obesity were older (mean difference: 1.05; 95%CI: 0.28 to 1.82; *p* < 0.01) with higher SBP (MD: 6.38; 95%CI: 3.64 to 8.07) and DBP (MD: 2.37; 95%CI: -0.36 to 4.88) when compared to controls. The characteristics of the included studies are presented in Table 1.

**Table 1:** Characteristics of studies reporting on the mean platelet volume in individuals with obesity (*n* = 13)

Author	Year	Country	No. participants	Primary aim	Main findings related to platelet indices	Instrument (Reference range)
Arslan [15]	2010	Turkey	47 Non-obese 56 Obese (BMI ≥ 27 kg/m^2^)	To investigate the MPV levels in obese adolescents. Moreover, to compare the MPV levels between patients with or without NAFLD and healthy controls	Obese adolescents had significantly higher MPV levels compared to healthy controls. In addition, the MPV is inversely associated with HDL cholesterol levels and platelet counts	EDTA, Sysmex XE-500 Reference range: 7.2–11.1 fL
Coban [17]	2005	Turkey	100 Non-obese (BMI < 26.6) 100 Obese (BMI ≥ 30 kg/m^2^)	To evaluate MPV in patients with obesity compared to non-obese controls	The MPV is increased in individuals with obesity when compared to the control group. In addition, the MPV was positively associated with BMI	Citrate, Abbot Cell-Dyn 3500
Coban [18]	2007	Turkey	30 Non-obese (BMI < 27.7) 30 Obese women (BMI ≥ 30 kg/m^2^)	To evaluate the effect of weight loss on the levels on the levels of MPV in obese patients	The MPV was higher in obese patients. Moreover, the MPV was directly associated with BMI	Citrate Abbot Cell Dyn 3500
Erdal [19]	2019	Turkey	45 Non-obese < 21 kg/m^2^) 45 Obese (BMI > 45 kg/m^2^)	To compare the complete blood counts, which included PCT and PLR values of healthy subjects with those of morbidly obese individuals in the young population	Platelet counts were increased in individuals with obesity. However, the MPV was comparable between lean and obese	NR
Esen [20]	2015	Turkey	204 Non-obese (BMI < 27.2) 290 Obese (BMI ≥ 30 kg/m^2^)	To compare the complete blood counts, which included PCT and PLR values of healthy subjects with those of morbidly obese individuals in the young population	The MPV was comparable between obese and non-obese individuals. Although the MPV was inversely correlated with BMI	EDTA, Sysmex XE-500 (Sysmex Corp, Kobe, Japan) Reference range: 7.2–11.1 fL
Furman-Niedzieko [21]	2014	Poland	129 Non-obese (BMI 21.3–25.7 kg/m^2^) 218 Obese (BMI > 25.1 kg/m^2^)	To assess the correlation between the platelet markers, especially the MPV, and the incidence of abdominal obesity in patients with the metabolic syndrome	The MPV was higher in individuals with obesity compared to controls	NR; SYSMEX XS-1000i
Furucouglu [24]	2016	Turkey	73 Non-obese (BMI < 25.53 kg/m^2^) 74 Obese (BMI ≥ 30–40 kg/m^2^)	To aim of our study was to evaluate the effect of weight and smoking status on CBC parameters and biomarkers	The MPV was comparable between obese and individuals with normal body weights. The BMI is positively correlated with platelet indices (Platelet distribution width, plateletcrit and platelet count) other than the MPV	NR
Hou [33]	2015	China	3527 Normal (BMI < 24 kg/m^2^) 899 Obese (BMI ≥ 28 kg/m^2^)	To investigate associations between adiposity indices and platelet indices	The MPV was in lower in obese compared non-obese individuals. The MPV levels were strongly associated with white cell count	EDTA and Citrate; CELL-DYN 3700, (Abbott, USA); Reference range: 7–11 fL
Ozkan [22]	2015	Turkey	48 Non-obese (BMI < 21 kg/m^2^) 60 Obese (BMI ≥ 23.7 kg/m^2^)	To investigate the relation between MPV levels and CIMT measurements	The MPV was increased in obese children with non-alcoholic fatty liver disease	NR
Pinto [23]	2019	Brazil	33 Non-obese (< 25 kg/m^2^) 69 Obese (> 30 kg/m^2^)	To verify the influence of EDTA on MPV among normal overweight and obese patients	The MPV was increased in individuals with obesity compared to controls. The BMI and waist circumference were positively associated with the MPV	K3-EDTA Cell-DYN Ruby analyser
Rihayi [32]	2018	Malaysia	56 Non-obese (BMI 18.5–22.9 kg/m^2^) 28 Obese (BMI 25.5-35 kg/m^2^)	To evaluate associations between markers of platelet activation and inflammation in men and women with varying body mass indices	The MPV was elevated in individuals with obesity when compared to controls. Moreover, the MPV was directly associated with the white cell count	EDTA; Sysmex KX-21
Tavil [34]	2006	Turkey	140 Non-obese 205 Obese (Abdominal obesity: > 102 cm in men; and > 88 cm in women)	to determine whether MPV values are increased in patients with MS, and secondly to evaluate the relationship between the severity of atherosclerosis and MPV patients with MS	The MPV was significantly increased in individuals with obesity	K3-EDTA Cell-DYN 3500 (Abbot, IL, USA) **Reference range:** 7.0–11.00 fL
Yilmaz[9]	2015	Turkey	16 Non-obese (BMI < 25 kg/m^2^) 25 Obese (BMI ≥ 25 kg/m	To compare hsCRP, MPV and NLR levels in lean and obese PCOS patients	The MPV is increased in individuals with obesity when compared to the control group. In addition, the MPV was positively associated with the BMI	Sysmex XE-2100

*BMI* Body Mass Index, *CBC* Complete blood count; *EDTA* ethylenediaminetetraacetic, *fL* Femtoliters, *HDL* High-density lipoprotein cholesterol, *K3-EDTA* tripotassium EDTA, *NAFLD* Non-Alcoholic fatty Liver Disease(NAFLD), *PCT* Plateletcrit, *PLR* Platelet leukocyte Ratio

**Table 2:** Cardiovascular-risk profile of included participants

**Effect Measure**	**Number of studies**	**Number of participants**	**Effect Estimate**					
			**Model**	**MD**	**SMD**	**95% CI**	**I**^**2**^**, *p*-value**	**Z, *p*-value**
BMI	10(16, 17, 19–23, 32, 34)	1902	RE	11.15	–	8.87 to 13.43	98%, *p* < 0.0001	9.59,*p* < 0.0001
Age	11(15, 17–23, 32, 34)	2004	FE	1.00	–	0.35 to 1.82	23%,*p* = 0.003	3.00, *p* = 0.0031
Platelet count	9(15–17, 19–24)	974	FE	-1.22		-2.42 to -1.65	41.93%,*p* = 0.09	-1.98,*p* = 0.047
**Blood Pressure**								
SBP	4(18, 20, 22, 34)	1007	RE	10.08	–	-0.22 to 20.37	97%, *p* < 0.0001	1.92, *p* = 0.06
DBP	3(18, 20, 22, 34)	1007	RE	4.60	–	-1.29 to 10.48	97%,*p* < 0.0001	4.60, *p* = 0.13
***Glucose metabolism***	6(17–20, 22, 34)	1297	FE	4.05	–	2.92 to 5.19	90%,*p* < 0.0001	7.00,*p* < 0.00001
***Lipid metabolism***								
Total cholesterol	7(16–22, 34)	1629	RE	–	0.19	0.00 to 0.38	68%,*p* = 0.005	2.00,*p* = 0.05
HDL Cholesterol	7(16–22, 34)	1629	RE	–	-0.68	-1.25 to -0.12	96%,*p* < 0.001	2.37,*p* = 0.02
LDL Cholesterol	7(16–22, 34)	1629	RE	–	0.20	0.00 to 0.41	71%,*p* = 0.002	2.00,*p* = 0.05
Triglycerides	7(16–22, 34)	1629	RE	–	0.60	0.20 to 1.01	93%,*p* < 0.001	2.90,*p* = 0.004

*BMI* Body mass index; *SBP* Systolic Blood Pressure; *DBP* Diastolic Blood Pressure; *HDL *high-density lipoprotein; *LDL* Low-density lipoprotein; *RE* Random-effects; *FE* Fixed effects

### Assessment of publication bias

There were 11 studies included in this meta-analysis, and visual inspection of the funnel plots indicated asymmetry. The contour-enhanced funnel plot was used to distinguish between publication bias and alternative sources of asymmetry. The funnel plot revealed that small studies were not only found in the regions of no statistical significance (*p* > 10%), but they also fell in the region of statistical significance (*p* < 5%). This suggests that asymmetry was a result of alternative sources but not solely a result of publication bias. In addition, we performed the Egger's regression test to explore the association between the reported effect size and the study size. In addition, there was no evidence of small study effects (*p* = 0.34) (Supplementary table 4S) or publication bias by the Egger test (bias: 0.17; *p* = 0.51).

### Quality assessment of the included studies

**Fig. 2:**
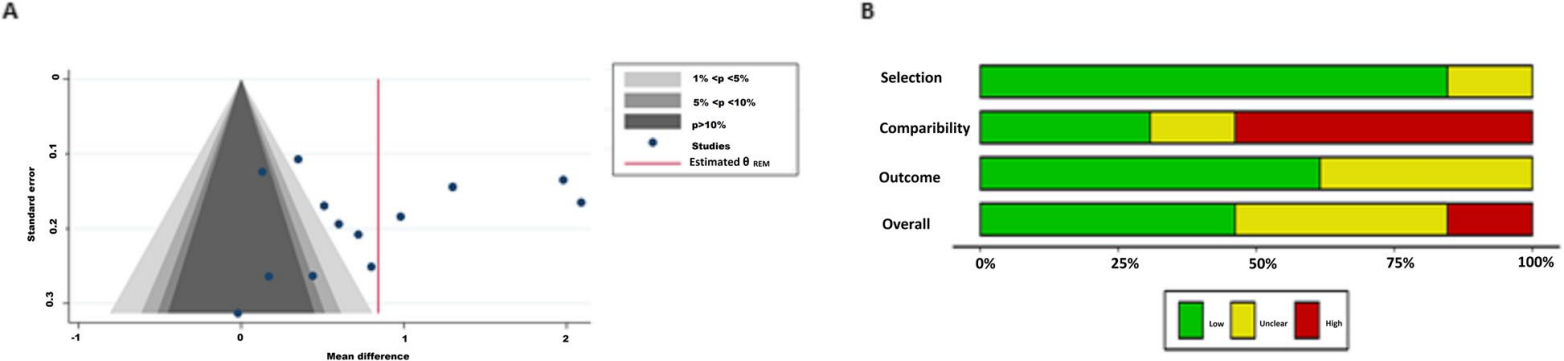
Publication and risk of bias assessment. **A** shows the funnel plots, and. **B** demonstrates the overall risk of bias

The Newcastle–Ottawa scale was used to assess the risk of bias in the included studies (Fig. 2B). In all, 42% of the included studies [15, 17, 18, 22, 34] scored as high-quality (scores > 7), and an equal proportion of 42% of the studies [16, 19, 20, 23, 32] were of moderate-quality (scores ranging from 5–6). While, only 16% were considered as having a low-quality [21, 24] and had scored lower than 5 (Table 2S).

#### Primary findings

A total of 7 studies reported increased MPV levels in children [15, 22] and adults with obesity [16–18, 21, 23]. Only three studies reported findings suggesting that the MPV remains unchanged in obesity [20, 24, 35]. Notably, the included studies reported an inverse [20] and a direct association [16–18, 23] between BMI levels and the MPV in individuals with obesity. Moreover, the MPV is also inversely associated with HDL cholesterol levels and platelet counts in individuals with obesity. The primary outcome of this meta-analysis included changes in the mean platelet volume in 814 individuals with obesity reported in 10 studies [15–20, 22–24, 36]. After pooling the effect estimates, we showed that the mean platelet volume significantly increased in individuals with obesity compared to non-obese individuals (MD 0.79; (95%CI: 0.42 to 1.16). However, the levels of statistical heterogeneity were high (I^2^ = 93.44%), which were unexplained by the test for subgroup analysis based on the risk of bias in the included studies (Fig. 2).

#### Secondary findings

In assessing the secondary outcome of the meta-analysis which focused on the ASCVD-risk in individuals with obesity. Only 5 (50%) of the included studies reported on fasting blood glucose levels [17–20, 22], and 6 studies [16–22] lipid profiles in individuals with obesity. As expected, our meta-analysis showed that individuals with obesity had higher; fasting blood glucose levels (MD:2.75; 95%CI, 1.55 to 3.94; I^2^ = 76%,*p* = 0.02); total cholesterol (SMD: 0.14 [95%CI: 0.03 to 0.25], I^2^ = 72%, *p* = 0.04); LDL cholesterol (SMD: 0.22[95%CI: -0.03 to 0.47], I^2^ = 76%, *p* = 0.009) and triglycerides (SMD: 0.43[95%CI:0.18 to 0.68], I^2^ = 75%, *p* = 0.0007) (Table 2).

### Subgroup and sensitivity analysis

**Fig. 3:**
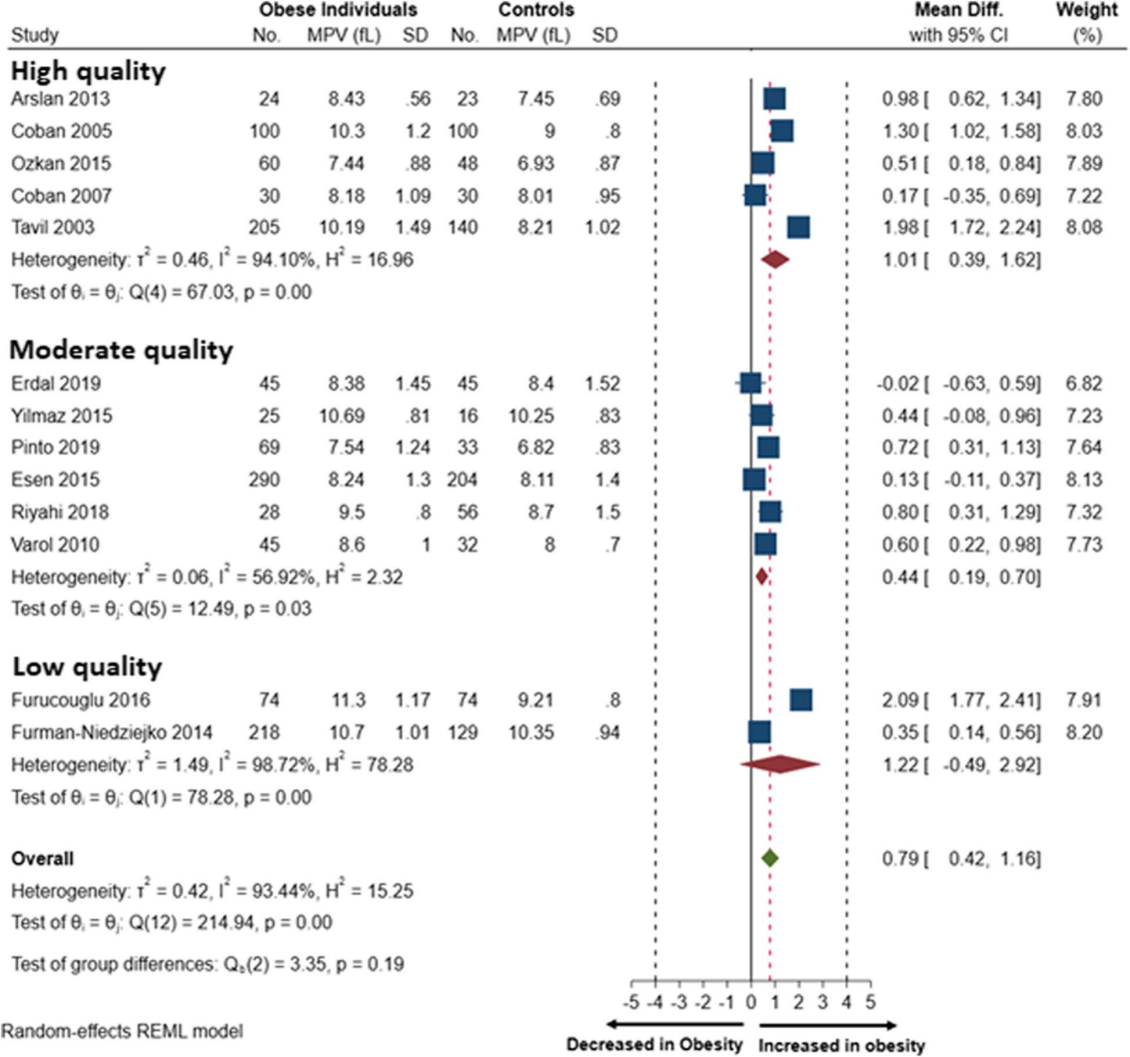
Forest plot showing the Random-effects pooled mean difference of the mean platelet volume in obese and non-obese individuals

There were substantial levels of statistical heterogeneity in the reported effect estimates. We, therefore, performed a subgroup analysis to explore the sources of heterogeneity based on the differences in the risk of bias in the included studies (Fig. 3). Although the small number of studies and covariance because of an unequal number of studies in each subgroup, the test for subgroup effect showed a significant subgroup effect (*p* = 0.19). This suggests that the reported differences in MPV may be influenced by the risk of bias in the included studies.

Notably, the forest plot (Fig. 3) demonstrates that the studies with high-quality showed a more significant mean difference (MD) in the MPV of individuals with obesity (MD: 1.01 [0.39 to 1.62]], I^2^ = 94.1%) when compared to studies with a moderate-quality (MD: 0.44 [0.19 to 0.70], I^2^ = 56.9%). In comparison, studies with a low-quality showed the highest increase in MPV in individuals with obesity compared to controls (MD: 1.22 [-0.49 to 2.92], I^2^ = 93.3%). Notably, amongst the studies reporting on a cohort of individuals with obesity from the Middle East, all studies were conducted in Turkey, and only 28.6%(*n* = 2) of the studies were moderate-quality. While the majority, 71% (*n* = 5) of the studies had a low-quality of bias (Table 1). Unlike the studies from Brazil and Poland, which were both of moderate-quality. The sensitivity analysis showed that studies with a low-quality overestimated the effect size of the primary outcome (0.98[0.33 to 1.62], I^2^ = 92.15%, *p* < 0.001) when compared to studies with a moderate-quality of bias (0.25[0.01 to 0.48], I^2^ = 62.06%, *p* < 0.041]. We also explored whether the variations in the included participants' age modified the effect estimate of the primary outcome (supplementary table 2S). Two of the included studies reported on the MPV of children (< 18 years of age) [15, 22], while the majority of the studies reported on cohorts comprised of adults (> 18 years of age) [16–21, 23, 24].

### Meta-regression analysis

We explored the associations between the markers of ASCVD-risk reported pooled mean platelet volume estimates. In the reported markers, collinearity existed between the BMI, systolic blood pressure, diastolic blood pressure and fasting blood glucose levels. So, the explanatory variables selected for the meta-regression model included Age, BMI, platelet counts, cholesterol and Triglyceride levels. Notably, the age of the study participants was reported in 8 studies [17–22], and BMI levels were reported in 7 studies [6–12]. Whereas only six studies [16–22] reported on the total cholesterol levels (Table 2).

In the meta-analysis, the levels of MPV were significantly elevated in obesity with moderate levels of certainty (Table 3). Interestingly, the meta-regression showed that age, BMI, and total cholesterol levels are significant confounders of the reported differences in changes in MPV in individuals with obesity (Table 2). While the inverse association between the BMI and MPV (Coefficient: -0.57, standard error (SE): 0.18, *p* < 0.001) is congruent with the previous study by Esen et al. [20], these contradict the positive associations reported in four of the included studies [16, 17, 22, 23]. Furthermore, the meta-regression showed a significant direct association between the differences in triglyceride and MPV levels (Coefficient: 4.99, standard error (SE): 1.14, *p* < 0.001).

**Table 3:** Summary of findings

**Mean platelet volume in individuals with obesity compared to non-obese individuals**						
**Patient or population**: Class I and Class II Obesity **Setting**: Outpatient **Intervention**: None **Comparison**: Individuals with Normal Body weights						
Outcomes	Absolute effects^*^ (95% CI)		Relative effect (95% CI)	№ of participants (studies)	Certainty of the evidence (GRADE)	Comments
	**Basal**	**SMD**				
Mean Platelet Volume (MPV) Scale from: 6.5 to 12.0	**8.5** (7.49–9.51)	**0.79** (0.42–1.16)	**Higher** (0.42 higher to 1.16 higher) -	1577 (9 observational studies)	⨁⨁⨁◯ MODERATE^a^	The MPV is increased in individuals with obesity when compared to non-obese individuals
**GRADE Working Group grades of evidence** **High certainty:** We are very confident that the true effect lies close to that of the estimate of the effect **Moderate certainty:** We are moderately confident in the effect estimate: The true effect is likely to be close to the estimate of the effect, but there is a possibility that it is substantially different **Low certainty:** Our confidence in the effect estimate is limited: The true effect may be substantially different from the estimate of the effect **Very low certainty:** We have very little confidence in the effect estimate: The true effect is likely to be substantially different from the estimate of effect						

*CI* Confidence interval; *SMD* Standardised mean difference

^*^The risk in the intervention group (and its 95% confidence interval) is based on the assumed risk in the comparison group and the relative effect of the intervention (and its 95% CI)

## Discussion

This meta-regression and meta-analysis is the first to estimate the MPV in individuals with obesity by pooling data reported in studies with a primary or secondary aim of evaluating the MPV in obese populations. The risk of bias in included studies was low to moderate, with only 15% of the studies deemed high-risk. In addition, the quality of the evidence was moderate for the primary outcome aimed at providing an estimate of the MPV in individuals with obesity. In comparison, the quality of evidence regarding the secondary outcome of ASCVD-risk in individuals with obesity was high. The MPV remains a cost-effective measure of platelet activation, which is under-utilised in the clinical setting. The primary reasons include methodological reasons and a lack of definitive reference intervals associated with clinical significance or outcomes. In our study, the majority of the included studies reported on EDTA collected blood (46%)[15, 20, 23, 32–34], while only 15% reported on citrated whole blood samples [17, 18] and 23% of the included studies failed to report on the anticoagulant used [19, 22, 24]. Although geographic and gender-specific differences exist for several haematological parameters, the included studies reported on similar MPV reference intervals ranging from 7.0- 11.1 fL [15, 20, 33, 34]. This may further support the comparability of the clinical significance of MPV associated findings across the included studies.

Our primary findings demonstrate the MPV is moderately higher (MD: 0.79) in individuals with obesity. Notably, substantial levels of statistical heterogeneity remained unexplained following a subgroup analysis based on the levels of potential bias and sensitivity analysis based on the age of the included participants. The secondary outcome of this study was addressed by performing a meta-regression, aimed at assessing the associations between the MPV and ASCVD-risk in individuals with obesity. The reported pooled estimates in this meta-analysis are congruent with previous findings of elevated MPV in individuals with obesity [15, 17–19, 21–23, 32, 34]. Our findings support the well-described levels of platelet activation in obesity and mechanisms that involve morphological and functional changes as a consequence of low-grade inflammation in obesity.

Upon activation, platelets undergo degranulation and change shape from quiescent disks to spread spheres with an increased diameter which correlates to the measured MPV. Larger platelets are metabolically active and densely express fibrinogen receptors, increasing their functional capability and affinity to forming aggregates [37]. Increased platelet activation and aggregation levels are strongly associated with cardiovascular complications, and the MPV is a well-establish independent risk factor for myocardial infarcts [38, 39]. Since obesity is a significant risk factor for cardiovascular disease, the routine monitoring of the MPV of individuals with obesity may offer is a cost-effective valuable marker used in the thrombotic-risk stratification of individuals at an increased risk of ASCVD. Moreover, the MPV has been associated with insulin resistance and coronary artery disease in non-obese individuals, with lifestyle modifications attenuating insulin resistance and MPV levels in these individuals [40].

Our secondary findings from the meta-regression analysis aimed at evaluating the associations between the ASCVD-risk factors and the MPV showed a strong association between MPV and the differences in the ASCVD-risk profiles of individuals with obesity compared to controls. An elevated MPV is associated with coronary artery disease in patients with the metabolic syndrome [34]. Notably, the confounders that modify the reported MPV estimates in this meta-analysis included age, BMI, platelet count, total cholesterol, and triglycerides. The pooled ASCVD-risk estimates showed that the included studies reported on individuals with hallmark features of the metabolic syndrome (MetS). This was illustrated by the basal characteristics of elevated fasting blood glucose levels, low levels of high-density lipoproteins (HDL) and elevated triglycerides. In all, our findings may be applicable in the context of individuals with obesity and MetS. In that context, an inverse relationship between BMI and MPV was previously reported in large cross-sectional studies comprising 290 individuals with obesity [20] and 3827 Korean adults [41]. However, in smaller studies, contradictory findings of a direct [16–18] and no association between BMI and MPV have been reported [24]. Moreover, in adolescents with obesity, elevated MPV levels were associated with increased HDL levels and platelet counts [15].

In our meta-regression analysis, the mean differences in age were associated with a decreased MPV (Table 2). Unlike in our meta-analysis, which included obese adults with a mean age of 44 years, in elderly patients (≥ 75 years), age is an independent predictor of larger MPV and median values above 10.85 fl were associated with increased prevalence of coronary artery disease. In our meta-analysis, the total cholesterol levels were slightly higher (SMD: 0.19) in individuals with obesity and these inversely correlated with the MPV. In comparison, the triglyceride levels were moderately increased in individuals with obesity (*p* = 0.004) (Table 4). In addition, elevated triglyceride levels were associated with increased MPV in individuals with obesity. In contrast, Park et al*.* showed that the MPV was inversely associated with fasting triglyceride levels in non-obese individuals [42].

**Table 4:** Meta-regression of potential modifiers of the reported differences in MPV

Parameter	Coefficient	SE	Z-value	*p*-value	95%CI
Age	-6.51	1.15	-5.68	< 0.0001	-8.76 to -4.26
BMI	-0.57	0.18	-3.12	0.002	-0.93 to -0.21
Platelet count	-3.83	1.01	3.81	< 0.0001	-5.80 to-1.86
TC	-4.52	1.06	-4.24	< 0.0001	-6.61 to -2.43
TG	4.99	1.14	4.36	< 0.0001	2.75 to 7.24

*BMI* Body Mass Index; *SE* Standard Error; *TC* Total Cholesterol; *TG* Triglyceride

The strengths of the meta-analysis and meta-regression include the comprehensive search and levels of certainty with minimal risk of bias in the reported findings. Although the interpretation of these findings should be taken with caution, the geographic distribution of the included participants was largely restricted to European countries, with mainly studies reporting on individuals with obesity in Turkey. Hence the potential influence of geographic settings on the reported effect estimates could not be determined using a subgroup analysis based on geographic regions due to the few studies from South America and Asia included in this meta-analysis. This would result in misleading subgroup effects due to covariance because of an unequal number of studies in each subgroup. Lastly, all the studies included in the meta-analysis and meta-regression were cross-sectional studies, and these are known to overestimate the overall effect estimate. Future studies should focus on determining cut-off MPV values associated with primary cardiovascular events in individuals with obesity who may have variable responses to antiplatelet therapy. The MPV may be useful in predicting patients at risk of residual platelet reactivity due to increased levels of circulating larger and hyperactive platelets.

## Conclusion

This meta-analysis and meta-regression provide comprehensive evidence on increased MPV levels associated with increased triglyceride levels and inversely associated with BMI in individuals with obesity. Overall, the findings suggest that MPV may be a valuable rapid marker for the monitoring and risk-stratification of individuals with obesity who may be at risk of developing cardiovascular disease associated with an elevated MPV and hypertriglyceridemia.

## Supplementary Information

**Additional file 1.** Reporting checklist for meta-analysis of observational studies.

**Additional file 2:** **Table S1.** Search strategy used on the Medline database using the PubMed search engine. **Table S2. **Search strategy used on the Embase database using OVID interface. **Table S3.** Assessment of risk of bias using the Newcastle-Ottawa Scale. **Table S4.** Analysis of publication bias and of small-study effect in the included studies. **Table S5. **Sensitivity analysis of studies included in meta-analysis of studies reporting on the MPV in obese individuals

## Data Availability

The authors confirm that the data supporting the findings of this study are available within the article and its supplementary files.
