# Supplementary material for: The mean platelet volume and atherosclerotic cardiovascular-risk factors in adults with obesity: a systematic review and meta-analysis of observational studies
Source: BMC Nutr. 2022 May 16;8:47. doi: 10.1186/s40795-022-00541-8 (PMC9109381; doi:10.1186/s40795-022-00541-8)
Supplement: Supplementary file 1 — Additional file 1. Reporting checklist for meta-analysis of observational studies. [file 40795_2022_541_MOESM1_ESM.docx]

# Reporting checklist for meta-analysis of observational studies.

Based on the MOOSE guidelines.

## Instructions to authors

Complete this checklist by entering the page numbers from your manuscript where readers will find each of the items listed below.

Your article may not currently address all the items on the checklist. Please modify your text to include the missing information. If you are certain that an item does not apply, please write "n/a" and provide a short explanation.

Upload your completed checklist as an extra file when you submit to a journal.

In your methods section, say that you used the MOOSEreporting guidelines, and cite them as:

Stroup DF, Berlin JA, Morton SC, Olkin I, Williamson GD, Rennie D, Moher D, Becker BJ, Sipe TA, Thacker SB. Meta-analysis of observational studies in epidemiology: a proposal for reporting. Meta-analysis Of Observational Studies in Epidemiology (MOOSE) group. JAMA. 2000; 283(15):2008-2012.

|  |  | Reporting Item | Page Number |
| --- | --- | --- | --- |
| **Title** |  |  |  |
|  | [#1](https://www.goodreports.org/moose/info/#1) | Identify the study as a meta-analysis of observational research | 1 |
| **Abstract** |  |  |  |
|  | [#2](https://www.goodreports.org/moose/info/#2) | Provide a structured summary including, as applicable: background; objectives; data sources; study eligibility criteria, participants, and interventions; study appraisal and synthesis methods; results; limitations; conclusions and implications of key findings; systematic review registration number (From PRISMA checklist) | 2 |
| **Background** |  |  |  |
|  | [#3a](https://www.goodreports.org/moose/info/#3a) | Problem definition | 3 |
|  | [#3b](https://www.goodreports.org/moose/info/#3b) | Hypothesis statement | 3 |
|  | [#3c](https://www.goodreports.org/moose/info/#3c) | Description of study outcomes | 5 |
|  | [#3d](https://www.goodreports.org/moose/info/#3d) | Type of exposure or intervention used | 5 |
|  | [#3e](https://www.goodreports.org/moose/info/#3e) | Type of study designs used | 5 |
|  | [#3f](https://www.goodreports.org/moose/info/#3f) | Study population | 5 |
| **Methods** |  |  |  |
| Search strategy | [#4a](https://www.goodreports.org/moose/info/#4a) | Qualifications of searchers (eg, librarians and investigators) | 5 |
| Search strategy | [#4b](https://www.goodreports.org/moose/info/#4b) | Search strategy, including time period included in the synthesis and keywords | 5 |
| Search strategy | [#4c](https://www.goodreports.org/moose/info/#4c) | Effort to include all available studies, including contact with authors | 5 |
| Search strategy | [#4d](https://www.goodreports.org/moose/info/#4d) | Databases and registries searched | 4 |
| Search strategy | [#4e](https://www.goodreports.org/moose/info/#4e) | Search software used, name and version, including special features used (eg, explosion) | 4 |
| Search strategy | [#4f](https://www.goodreports.org/moose/info/#4f) | Use of hand searching (eg, reference lists of obtained articles) | 4 |
| Search strategy | [#4g](https://www.goodreports.org/moose/info/#4g) | List of citations located and those excluded, including justification | 6 |
| Search strategy | [#4h](https://www.goodreports.org/moose/info/#4h) | Method of addressing articles published in languages other than English | 4 |
| Search strategy | [#4i](https://www.goodreports.org/moose/info/#4i) | Method of handling abstracts and unpublished studies | 5 |
| Search strategy | [#4j](https://www.goodreports.org/moose/info/#4j) | Description of any contact with authors | N/A |
|  | [#5a](https://www.goodreports.org/moose/info/#5a) | Description of relevance or appropriateness of studies gathered for assessing the hypothesis to be tested | 5 |
|  | [#5b](https://www.goodreports.org/moose/info/#5b) | Rationale for the selection and coding of data (eg, sound clinical principles or convenience) | 5 |
|  | [#5c](https://www.goodreports.org/moose/info/#5c) | Documentation of how data were classified and coded (eg, multiple raters, blinding, and interrater reliability) | 5 |
|  | [#5d](https://www.goodreports.org/moose/info/#5d) | Assessment of confounding (eg, comparability of cases and controls in studies where appropriate) | 5 |
|  | [#5e](https://www.goodreports.org/moose/info/#5e) | Assessment of study quality, including blinding of quality assessors; stratification or regression on possible predictors of study results | 5 |
|  | [#5f](https://www.goodreports.org/moose/info/#5f) | Assessment of heterogeneity | 6 |
|  | [#5g](https://www.goodreports.org/moose/info/#5g) | Description of statistical methods (eg, complete description of fixed or random effects models, justification of whether the chosen models account for predictors of study results, dose-response models, or cumulative meta-analysis) in sufficient detail to be replicated | 6 |
|  | [#5h](https://www.goodreports.org/moose/info/#5h) | Provision of appropriate tables and graphics | 8-11 |
| **Results** |  |  |  |
|  | [#6a](https://www.goodreports.org/moose/info/#6a) | Graphic summarizing individual study estimates and overall estimate | 12 |
|  | [#6b](https://www.goodreports.org/moose/info/#6b) | Table giving descriptive information for each study included | 9-11 |
|  | [#6c](https://www.goodreports.org/moose/info/#6c) | Results of sensitivity testing (eg, subgroup analysis) | 12 |
|  | [#6d](https://www.goodreports.org/moose/info/#6d) | Indication of statistical uncertainty of findings | 16 |
| **Discussion** |  |  |  |
|  | [#7a](https://www.goodreports.org/moose/info/#7a) | Quantitative assessment of bias (eg. publication bias) | 17 |
|  | [#7b](https://www.goodreports.org/moose/info/#7b) | Justification for exclusion (eg, exclusion of non–English-language citations) | 17 |
|  | [#7c](https://www.goodreports.org/moose/info/#7c) | Assessment of quality of included studies | 17 |
| **Conclusion** |  |  |  |
|  | [#8a](https://www.goodreports.org/moose/info/#8a) | Consideration of alternative explanations for observed results | 18 |
|  | [#8b](https://www.goodreports.org/moose/info/#8b) | Generalization of the conclusions (ie, appropriate for the data presented and within the domain of the literature review) | 18 |
|  | [#8c](https://www.goodreports.org/moose/info/#8c) | Guidelines for future research | 18 |
|  | [#8d](https://www.goodreports.org/moose/info/#8d) | Disclosure of funding source | 18 |

None Reproduced with permission from JAMA. 2000. 283(15):2008-2012. Copyright © 2000 American Medical Association. All rights reserved.This checklist can be completed online using <https://www.goodreports.org/>, a tool made by the [EQUATOR Network](https://www.equator-network.org) in collaboration with [Penelope.ai](https://www.penelope.ai)
