# Supplementary material for: The mean platelet volume and atherosclerotic cardiovascular-risk factors in adults with obesity: a systematic review and meta-analysis of observational studies
Source: BMC Nutr. 2022 May 16;8:47. doi: 10.1186/s40795-022-00541-8 (PMC9109381; doi:10.1186/s40795-022-00541-8)
Supplement: Supplementary file 2 — Additional file 2: Table S1. Search strategy used on the Medline database using the PubMed search engine. Table S2. Search strategy used on the Embase database using OVID interface. Table S3. Assessment of risk of bias using the Newcastle-Ottawa Scale. Table S4. Analysis of publication bias and of small-study effect in the included studies. Table S5. Sensitivity analysis of studies included in meta-analysis of studies reporting on the MPV in obese individuals [file 40795_2022_541_MOESM2_ESM.docx]

**Online supplementary data**

**Title: The mean platelet volume and atherosclerotic cardiovascular-risk in adults with obesity: A meta-analysis of observational studies**

Nkambule Bongani B^1^, Mxinwa Vuyolwethu^1^, Nyambuya Tawanda M^1^, Dludla Phiwayinkosi V^1^.

**Supplementary Tables**

**Table S1.** PubMed search strategy

**Table S2**. Assessment of risk of bias using the Newcastle-Ottawa Scale.

**Table S3**. Publication bias and analysis of small-study effect in the included studies

**Table 4S.** Sensitivity analysis of studies included in meta-analysis of studies reporting on the MPV in obese individuals

**Corresponding author:** Bongani B. Nkambule, School of Laboratory Medicine and Medical Sciences (SLMMS), College of Health Sciences, University of KwaZulu-Natal, Durban, South Africa. Private Bag X54001, Durban, 4000

**Table S1.** Search strategy used on the Medline database using the PubMed search engine

|  | "Obesity"[Mesh] AND (("blood platelets"[MeSH Terms] OR ("blood"[All Fields] AND "platelets"[All Fields]) OR "blood platelets"[All Fields] OR "platelet"[All Fields]) AND ("physiology"[Subheading] OR "physiology"[All Fields] OR "function"[All Fields] OR "physiology"[MeSH Terms] OR "function"[All Fields])) OR "Mean Platelet Volume"[Mesh] |
| --- | --- |
| AND | “Clinical Study[ptyp]” |
| AND | "loattrfull text"[sb] |
| AND | (“Humans” [Mesh]) |

**Table S2.** Search strategy used on the Embase database using OVID interface

| **#** | **Search term** | **Results** |
| --- | --- | --- |
| 1 | Obesity.mp. [mp=title, abstract, full text, caption text] | 330458 |
| 2 | Blood platelets.mp. [mp=title, abstract, full text, caption text] | 4322 |
| 3 | Thrombocytes.mp. [mp=title, abstract, full text, caption text] | 4098 |
| 4 | platelet count.mp. [mp=title, abstract, full text, caption text] | 83330 |
| 5 | mean platelet volume.mp. [mp=title, abstract, full text, caption text] | 3212 |
| 6 | plateletcrit.mp. [mp=title, abstract, full text, caption textplatelet distribution width.mp. [mp=title, abstract, full text, caption text] | 703 |
| 7 | 1 and 2 | 254 |
| 8 | 1 and 3 | 211 |
| 9 | 1 and 4 | 4785 |
| 10 | 1 and 5 | 425 |
| 11 | 1 and 6 | 32 |
| 12 | 1 and 7 | 98 |
| 13 | 5 and 8 | 13 |

**Table S3**. Assessment of risk of bias using the Newcastle-Ottawa Scale.

|  | | **Selection** | | | | |  | | **Comparability** | | **Outcome** | | | **Total** |  |
| --- | --- | --- | --- | --- | --- | --- | --- | --- | --- | --- | --- | --- | --- | --- | --- |
| **Study ID** | **Representative of obese patients** | | **Selection of control** | **Sample Size** | **Diagnose or criteria of classifying obesity** |  | | **Based on the study design or analysis.** | | **Ascertainment of the method** | | **Statistical test** |  | |  |
| ***Cross-Sectional studies*** |  | |  |  |  |  | |  | |  | |  |  | |  |
| Arslan et al., 2013 | * | | * | - | *** |  | | ** | | ** | | * | 10 | |  |
| Coban et al., 2005 | * | | * | - | *** |  | | ** | | ** | | * | 10 | |  |
| Coban et al., 2007 | * | | * | * | *** |  | | ** | | ** | | * | 11 | |  |
| Erdal et al., 2019 | * | | * | - | ** |  | | ** | | * | | * | 8 | |  |
| Esen et al., 2015 | * | | * | - | *** |  | | ** | | * | | * | 9 | |  |
| Furman-Niedziejko et al, 2014 | * | | - | - | * |  | | ** | | * | | - | 5 | |  |
| Furucouglunglo et al, 2016 | * | | * | - | * |  | | * | | * | | * | 6 | |  |
| Ozkan et al, 2015 | * | | * | - | *** |  | | ** | | ** | | * | 10 | |  |
| Pinto et al, 2019 | - | | - | - | *** |  | | - | | - | | * | 4 | |  |
| Rihayi et al, 2018 | * | | * | - | *** |  | | - | | ** | | * | 8 | |  |
| Tavil et al, 2007 | ** | | * | * | *** |  | | ** | | ** | | * | 12 | |  |
| Yilmaz et al, 2015 | * | | * | - | *** |  | | ** | | * | | * | 9 | |  |
|  |  | |  |  |  |  | |  | |  | |  |  | |  |
| ***Cohort study*** |  | |  |  |  |  | |  | |  | |  |  | |  |
| Hou et al, 2015 | ** | | * | - | *** |  | | ** | | ** | | * | 11 | |  |

*each star is allocated a single point in the total column. The higher the score the lower the risk of bias.

| **Outcome** | **Estimate** | **SE** | **Z-value** | **p-value** | **No. of studies** |
| --- | --- | --- | --- | --- | --- |
| MPV | 1.04 | 2.63 | 1.04 | 0.3241 | 10 |
| *Test of H_0_: no small-study effects* | |  |  |  |  |

**Table S4** . Analysis of publication bias and of small-study effect in the included studies

**Table S5.** Sensitivity analysis of studies included in meta-analysis of studies reporting on the MPV in obese individuals

| **Parameter** | **Number of studies (n)** | **Studies omitted** | **Hedge’ g (95%CI)** | **I^2^ (%), p-value** | **Test for subgroup effect:**  **Chi^2^, p-value** |
| --- | --- | --- | --- | --- | --- |
| **Age** |  |  |  |  | 0.59,p=0.442 |
| Children (>18) | n=2 (1,2) | n=8 (3–10) | 1.022[0.085 to 1.96] | 84.12%,p<0.001 |  |
| Adults (<18) | n=8 (3–10) | n=2 (1,2) | 0.602[0.084 to 1.12] | 95.10%, p=0.442 |  |
|  |  |  |  |  |  |

**References**

1. Arslan N, Makay B. Mean Platelet Volume in Obese Adolescents with Nonalcoholic Fatty Liver Disease. 2010;i:807–13.

2. Özkan EA, Khosroshahi HE, Serin H, Özdemir ZT, Kılıç M, Ekim M, et al. The evaluation of carotid intima-media thickness and mean platelet volume values and correlation with cardiac functions in obese children. Int J Clin Exp Med. 2015;8(12):22557–63.

3. Erdal E, Inanir M. Platelet-to-lymphocyte ratio (PLR) and Plateletcrit (PCT) in young patients with morbid obesity. Rev Assoc Med Bras. 2019;65(9):1182–7.

4. Yilmaz MA, Duran C, Basaran M. The mean platelet volume and neutrophil to lymphocyte ratio in obese and lean patients with polycystic ovary syndrome. J Endocrinol Invest. 2016;39(1):45–53.

5. Pinto RVL, Rodrigues G, Simões RL, Porto LC. Analysis of Post-Sample Collection EDTA Effects on Mean Platelet Volume Values in Relation to Overweight and Obese Patient Status. Acta Haematol. 2019;142(3):149–53.

6. Furuncuoǧlu Y, Tulgar S, Dogan AN, Cakar S, Tulgar YK, Cakiroglu B. How obesity affects the neutrophil/lymphocyte and platelet/lymphocyte ratio, systemic immune-inflammatory index and platelet indices: A retrospective study. Eur Rev Med Pharmacol Sci. 2016;20(7):1300–6.

7. Furman-Niedziejko A, Rostoff P, Rychlak R, Golinska-Grzybala K, Wilczynska-Golonka M, Golonka M, et al. Relationship between abdominal obesity, platelet blood count and mean platelet volume in patients with metabolic syndrome. Folia Med Cracov. 2014;54(2):55–64.

8. Esen B, Atay AE, Gunoz N, Gokmen ES, Sari H, Cakir I, et al. The relation of mean platelet volume with microalbuminuria and glomerular filtration rate in obese individuals without other metabolic risk factors: The role of platelets on renal functions. Clin Nephrol. 2015;83(6):322–30.

9. Coban E, Ozdogan M, Yazicioglu G, Akcit F. The mean platelet volume in patients with obesity. Int J Clin Pract. 2005;59(8):981–2.

10. Coban E, Yilmaz A, Sari R. The effect of weight loss on the mean platelet volume in obese patients. 2007;i(May):212–6.
